# Sex-differences in anxiety, neuroinflammatory markers, and enhanced fear learning following chronic heroin withdrawal

**DOI:** 10.1007/s00213-023-06310-0

**Published:** 2023-01-12

**Authors:** Shveta V. Parekh, Lydia O. Adams, Gillian A. Barkell, Donald T. Lysle

**Affiliations:** Department of Psychology and Neuroscience, University of North Carolina at Chapel Hill, CB#3720, Chapel Hill, NC 27599-3270 USA

**Keywords:** Opioid, Withdrawal, PTSD, Sex differences, Anxiety, EPM, Enhanced fear learning

## Abstract

Post-traumatic stress disorder (PTSD) and opioid use disorder (OUD) are comorbid in clinical populations. However, both pre-clinical and clinical studies of these co-occurring disorders have disproportionately represented male subjects, limiting the applicability of these findings. Our previous work has identified chronic escalating heroin administration and withdrawal can produce enhanced fear learning. This behavior is associated with an increase in dorsal hippocampal (DH) interleukin-1β (IL-1β), tumor necrosis factor-α (TNF-α), and glial fibrillary acidic protein (GFAP) immunoreactivity. Further, we have shown that these increases in IL-1β and TNF-α are mechanistically necessary for the development of enhanced fear learning. Although these are exciting findings, this paradigm has only been studied in males. The current studies aim to examine sex differences in the behavioral and neuroimmune effects of chronic heroin withdrawal and future enhanced fear learning. In turn, we determined that chronic escalating heroin administration can produce withdrawal in female rats comparable to male rats. Subsequently, we examined the consequence of heroin withdrawal on future enhanced fear learning and IL-1β, TNF-α, and GFAP immunoreactivity. Strikingly, we identified sex differences in these neuroimmune measures, as chronic heroin administration and withdrawal does not produce enhanced fear learning or immunoreactivity changes in females. Moreover, we determined whether heroin withdrawal produces short-term and long-term anxiety behaviors in both female and males. Collectively, these novel experiments are the first to test whether heroin withdrawal can sensitize future fear learning, produce neurobiological changes, and cause short-term and long-term anxiety behaviors in female rats.

## Introduction

Post-traumatic stress disorder (PTSD) and opioid use disorder (OUD) are highly comorbid in clinical populations, as nearly 35% of individuals with an opioid use disorder have comorbid PTSD (Saha et al. 2016). Further, this comorbidity leads to worsened outcomes in several areas of social, occupational, and emotional functioning, as well as in disorder duration and treatment outcomes when compared to outcomes of either disorder alone (Mills et al. 2018). However, both the pre-clinical and clinical study of these co-occurring disorders has disproportionately focused on male subjects, limiting the applicability and scope of these findings. Not only are females underrepresented as research subjects, men and women also display divergent symptomatic presentation, course, and treatment outcomes of both PTSD and OUD. In turn, the neurobiological mechanisms underlying the sex-differences of this comorbidity represent an important area of study, paving the way for improved diagnostics and therapeutics that effectively treat both men and women.

When examining the rates of substance use disorder comorbidity, a national epidemiological study found that 27.9% of women and 51.9% of men with lifetime PTSD also had a substance use disorder (SUD) (Mills et al. 2018). Although there are lower PTSD-OUD comorbidity rates for women than men, the lifetime prevalence of PTSD among women (9.7%) is 2.5 times that of men (3.6%), and the 12-month prevalence for women (5.2%) is nearly three times that of men (1.8%) (Kessler et al. 1995). The higher frequency of PTSD in women can be explained to a certain extent by the difference in traumatic events women tend to experience, as well as the prevalence of trauma, differences in stress-coping, and higher levels of associated risk factors such as depression and neuroticism. However, these factors do not entirely account for observed sex differences in PTSD (Christiansen and Hansen 2015). Regardless of trauma type or prevalence, women experience more chronic PTSD and different symptoms and comorbidities than men, which suggests that neurobiological sex differences underlie this occurrence (Pooley et al. 2018).

To study this PTSD-OUD comorbidity, we developed an animal model for chronic escalating heroin administration and withdrawal that we use in combination with an enhanced fear learning (EFL) paradigm (Parekh et al. 2020; Parekh et al. 2021). Animals first receive heroin for ten days followed by a spontaneous withdrawal, and then undergo the EFL paradigm. In the EFL paradigm, rats previously exposed to a severe stressor show an exaggerated or enhanced fear response to a mild form of stress in a separate, distinct context. Although it is difficult to incorporate all the symptoms of PTSD into a preclinical animal model, EFL effectively demonstrates hyperarousal and greater susceptibility to future fear learning, a prominent component of human PTSD. The hyperarousal and enhanced reactivity response captured using the enhanced fear learning paradigm offers the opportunity to investigate critical symptoms of clinical PTSD. The combination of chronic escalating heroin administration and withdrawal and EFL has been extensively studied with male rats and reliably produces withdrawal behaviors as well as enhanced fear learning and long-lasting hyperarousal in the EFL paradigm (Parekh et al. 2020; Parekh et al. 2021). In addition to these striking behavioral consequences, we have demonstrated that heroin withdrawal induces neurobiological changes in male animals, specifically, increases in IL-1β, TNF-α, and GFAP immunoreactivity within the dorsal hippocampus (Parekh et al. 2020; Parekh et al. 2021). Further, we have also shown that these enhanced levels of pro-inflammatory cytokines following heroin withdrawal play a mechanistic role in the development of enhanced fear learning. For example, infusions of IL-1RA, an IL-1 receptor antagonist, into the dorsal hippocampus following heroin withdrawal prevented the development of enhanced fear learning (Parekh et al. 2020). Infusion of etanercept, a TNF-α inhibitor, following heroin withdrawal also inhibited enhanced fear learning development (Parekh et al. 2021). However, these studies have not been conducted in females, necessitating the heroin withdrawal EFL paradigm to be tested in female animals in order to examine potential sex differences in withdrawal, EFL behavior, and neuroimmune changes.

The current studies aim to examine sex differences in the behavioral and neuroimmune effects of chronic heroin withdrawal and future enhanced fear learning. To this end, experiment 1 determined whether chronic escalating heroin can produce withdrawal in females. We identified that chronic escalating heroin administration does indeed produce a reliable heroin withdrawal comparable to male rats. Subsequently, experiment 2 examined the consequence of heroin withdrawal on future enhanced fear learning, and strikingly, chronic heroin administration and withdrawal does not produce enhanced fear learning in females. To determine the consequences of heroin withdrawal on generalized anxiety, experiment 3 identified that 24 h following heroin withdrawal both males and females developed elevated levels of anxiety behaviors in the elevated plus maze (EPM); however, 7 days following withdrawal, only males exhibited these symptoms. Lastly, experiment 4 tested whether heroin withdrawal affects IL-1β, TNF-α, and GFAP immunoreactivity in females. Collectively, these novel experiments are the first to test whether heroin withdrawal can sensitize future fear learning, produce neurobiological changes, and cause short-term and long-term anxiety behaviors in female rats.

## Materials and methods

### Animals

Adult female and male Sprague–Dawley rats (225–250 g, Charles River Laboratories, Raleigh, NC, USA) were individually housed under a reversed 12-h light/dark cycle. Rats were given ad libitum access to food and water and were regularly handled throughout experimentation. All procedures were conducted with approval from the University of North Carolina at Chapel Hill Institutional Animal Care and Use Committee.

### Drug administration

Heroin (diacetylmorphine hydrochloride, National Institute on Drug Abuse (NIDA) Drug Supply Program, Bethesda, MD, USA) was dissolved in sterile 0.9% saline to produce 1.0, 2.5, 5.0, 7.5, or 10.0 mg/mL solutions and stored at 4 °C until time of injection.

### Chronic escalating heroin administration and withdrawal

**Fig. 1:**
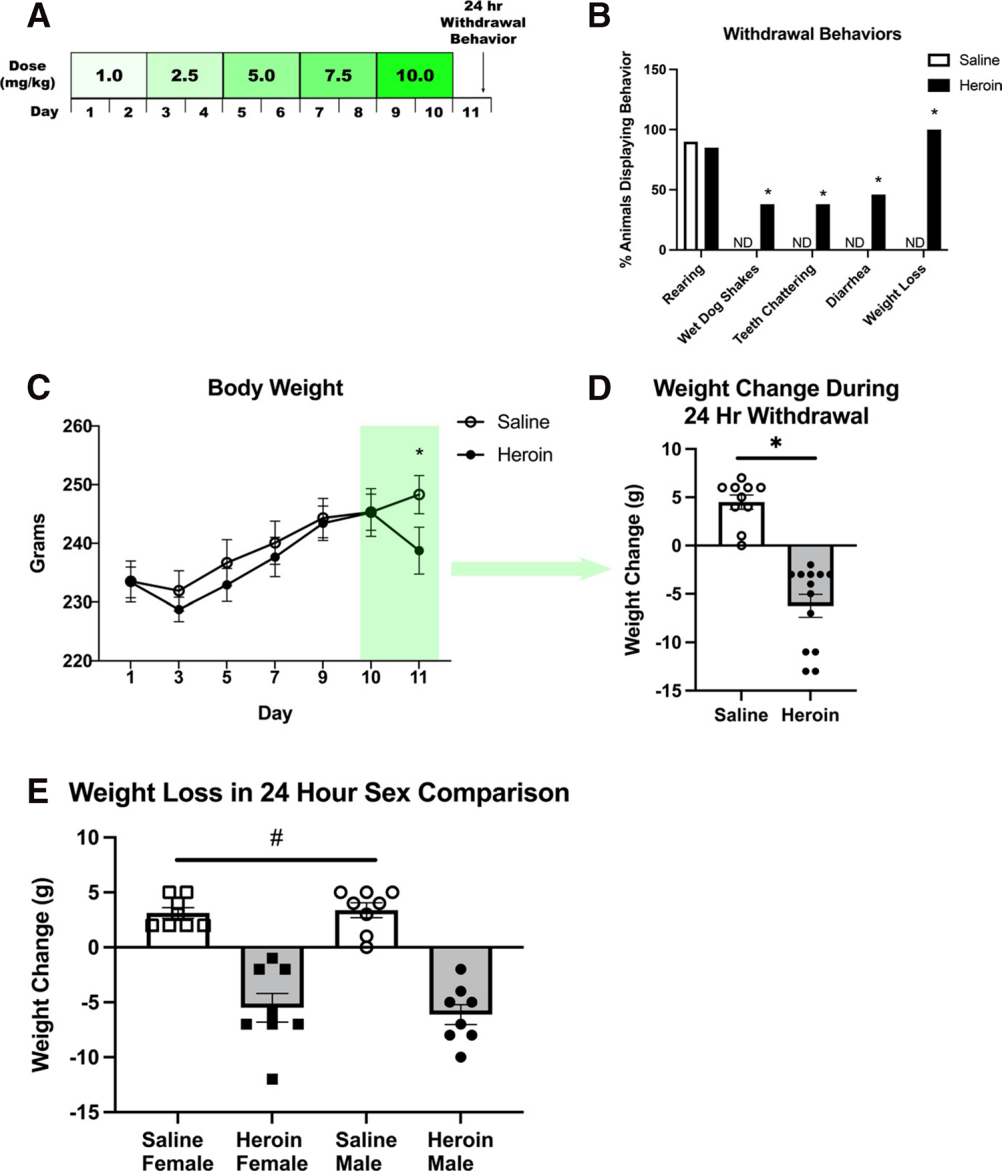
Chronic escalating heroin administration produces withdrawal behaviors in females. Chronic escalating heroin administration schematic (**A**). Withdrawal behaviors (**B**). Overall body weight across days of heroin administration (**C**) and weight change in 24-h withdrawal (*N* = 23, *n* = 10–13) (**D**). Sex comparison in weight loss during 24-h withdrawal (*N* = 32, *n* = 7–8) (**E**). *, statistically significant difference relative to respective control. #, statistically significant main effect of heroin, ND, not detected. Error bars indicate SEM

Animals were randomly assigned to drug (heroin or saline) treatment and heroin withdrawal timepoint (0, 24, 48, or 72 h) and underwent chronic escalating heroin administration as described previously (Parekh et al. 2020; Parekh et al. 2021). Briefly, rats were injected with heroin or saline 3 times daily (subcutaneous, s.c.) over 24 h periods for 10 days, with a dose increase every other day: 3.0 (3 × 1.0) mg/kg/day on days 1–2, 7.5 (3 × 2.5) mg/kg/day on days 3–4, 15.0 (3 × 5.0) mg/kg/day on days 5–6, 22.5 (3 × 7.5) mg/kg/day on days 7–8, and 30 (3 × 10) mg/kg/day on days 9–10 (Fig. 1a). Animal weights were measured on every dose increase day and subsequent withdrawal timepoints. This chronic escalating and withdrawal paradigm robustly produces withdrawal at the 24-h time point indicating both dependence of drug and subsequent withdrawal (Zhou et al. 2013; Parekh et al. 2020; Parekh et al. 2021).

### Chronic heroin and withdrawal-enhanced fear learning

This procedure has been previously described at length (Parekh et al. 2020; Parekh et al. 2021). Briefly, animals underwent chronic escalating heroin administration and withdrawal in their home cage. Seven days after the start of withdrawal, animals were placed into a novel context for 15 min of habituation. On day 8, animals were placed into the same context for a single scrambled foot shock (1 mA, 1 s) at 3 min and 12 s. On days 9, 10, 15, and 22 (test days 1, 2, 7, and 14), animals were placed into the same context for 8 min and 32 s and behavior was recorded to measure freezing behavior, a measure of learned fear (Fig. 3a). The Ethovision XT video tracking software (Noldus Information Technology Inc.) was used to analyze freezing behavior. The activity analysis feature (activity threshold = 10) was used to calculate the percent of time each animal was inactive during each contextual fear test and at baseline. Weight was measured at each timepoint to determine withdrawal-induced weight change. Following experimentation, the animals were sacrificed via rapid cervical dislocation.

### Elevated plus maze (EPM)

Anxiety-like behaviors were measured in the EPM. Testing was conducted during the dark cycle at the 24-h and 7-day withdrawal time point. Each session lasted 5 min and was conducted under lights-off conditions. A red-light lamp was used for visibility during the session. The EPM was elevated 50 cm above the floor and consisted of a central part (5 cm × 5 cm), two opposing open arms (30 cm × 5 cm), and two opposing closed arms (30 cm × 5 cm) with a 15-cm height (Harvard Apparatus, Holliston, MA, USA). Each animal was placed in the central part of the maze facing an open arm at the beginning of the session. The maze was cleaned with 75% ethanol and dried between each use. Exploratory behavior on the maze was filmed with a GoPro HERO10 camera, and later measured by an observer blind to experimental condition. Open/closed arm entries, open/closed arm time, and stretch attend postures were measured.

### Vaginal lavage and histology

Estrous cycle was assessed during the experiment at the following time points: 24-h withdrawal time point, B-shock, and test days 1, 2, 7, and 14. During assessment, the animals were restrained and the animal’s tail was elevated to visualize the vagina. The vaginal cells were flushed by gently introducing approximately 100 μl of saline using a sterile transfer pipette. The pipette was placed at the entrance of the vaginal canal, and saline was slowly released into vagina and then drawn back into the tip about 4 to 5 times in the same pipette. The fluid was then introduced on a glass slide, air-dried, and stained using a Wright Giemsa Stain, modified (Sigma-Aldrich) method. Vaginal cell distribution was examined using a Leica DM 6000B microscope (Leica Microsystems, Buffalo Grove, IL, USA) and estrous phase (diestrus, proestrus, estrus, or metestrus) was determined according to methods described by Cora and colleagues (Cora et al. 2015).

### Tissue collection and histology

Animals were sacrificed by transcardial perfusion 24-h into withdrawal. Animals were terminally anesthetized with 9:1 (vol/vol) ketamine hydrochloride (100 mg/mL) mixed with xylazine (100 mg/mL), and transcardially perfused with ice-cold phosphate buffer (PB; pH = 7.4) followed by 4% paraformaldehyde in 0.1 M PB. Brains were extracted and post-fixed in 4% paraformaldehyde for 6 h, and used 30% sucrose for cryoprotection with 0.1% sodium azide at 4 °C. Once the brains were saturated with sucrose, brains were cut into 40-μm coronal sections on a cryostat (Leica CM 3050 S, Leica Microsystems, Buffalo Grove, IL, USA).

### Immunohistochemistry

Fluorescent immunohistochemistry (IHC) was used to examine alterations in dorsal hippocampus (DH) TNF-α, IL-1β, and GFAP in the dentate gyrus (DG) at the 24-h heroin withdrawal time point. The IHC protocol used here has been described previously (Jones et al. 2015; Parekh et al. 2020; Parekh et al. 2021). Briefly, tissue sections were washed three times for 10 min in 0.1 M phosphate buffer (PB, pH = 7.4), followed by a 1-h incubation in 5% normal goat serum (NGS) and 0.5% TritonX100 in 0.1 M PB at room temperature. The following primary antibodies were used: rabbit anti-TNF alpha (1:1000, Abcam, Cambridge, MA, Cat# ab66579), rabbit anti-IL-1β (1:500, Abcam, Cambridge, MA, cat. no. Ab9722, USA), mouse anti-GFAP (1:1000, ThermoFisher Scientific, Waltham, MA, cat. no. MS-1376P, USA). Tissue sections were incubated in each primary antibody, 5% NGS, and 0.5% TritonX100 in 0.1 M PB overnight at 4 °C and washed three times for 10 min in 0.1 M PB. To visualize TNF-α, IL-1β, and GFAP, tissues were incubated in the following Alexa Fluor conjugated secondary antibodies: goat anti-rabbit 488 (1:1000, ThermoFisher Scientific, Waltham, MA, cat. no. A11008) and goat anti-mouse 594 (1:1000, ThermoFisher Scientific, Waltham, MA, cat. no. A11005). Tissue sections were incubated in each secondary antibody, 5% NGS, and 0.5% TritonX100 in 0.1 M PB for 1-h at room temperature. Primary antibodies were verified by no primary control stains. Sections were mounted onto SuperFrost Plus slides (Fisher Scientific, Pittsburgh, PA) using Vectashield with DAPI hardset mounting medium (Vector Laboratories, Burlingame, CA).

### Microscopy

Fluorescent microscopy (Leica DM6000 B widefield light microscope, Leica Microsystems, Buffalo Grove, IL, USA) was used to capture color images. The microscopy protocol used here has been described previously (Parekh et al. 2020; Parekh et al. 2021). Positive fluorescence in images was quantified using automatic ImageJ (NIH) triangle thresholding feature. The implementation of the ImageJ automatic triangle algorithm has been previously described (Zack et al. 1977). Briefly, the algorithm assumes a maximum peak near one end of the histogram and searches for intensity toward the end of the histogram bins. Three to five sections were analyzed bilaterally per animal for the dorsal dentate gyrus and values were averaged and expressed as percent positive stain. In addition, the number of TNF-α, GFAP, and IL-1β positive cells overlaid with DAPI, an indicator of cellular nuclei, was counted manually in all the images taken. All analyses including thresholding and counting were made blind to treatment condition. Tissue from perfusions that yielded high nonspecific background which interfered with thresholding was dropped from the analysis. In these perfusions, gross inspection of the brain did not reveal a fixed appearance—void of blood in circulatory system (white to pale yellow color), but instead was found to be reddish, indicative of blood present. Moreover, automatic thresholding failed on this tissue and resulted in exclusion from analysis. The decision to exclude these samples was made blind to the treatment group. Publication images were compiled with the Adobe Photoshop CS software (Creative Cloud Photoshop v22.1, San Jose, CA, USA). Color levels and background were reduced for optimal representation with level tools. Images from all experimental groups were treated equally.

### Statistical analysis

In experiment 1, withdrawal behaviors were analyzed using a *χ*^2^ test. Body weight was analyzed using a 2 (Drug Treatment) × 7 (Day) repeated measures ANOVA. Weight change and withdrawal composite score were analyzed using an unpaired, two-tailed Student’s *t* test. Weight change sex comparison was analyzed using a 2 (Drug Treatment) × 2 (Sex) ANOVA. In experiment 2, a two-way (Drug Treatment, Sex) ANOVA was used to analyze baseline freezing data. A 2 (Drug Treatment) × 2 (Sex) × 4 (Test Day) ANOVA was used to analyze freezing behavior across test days between sexes. Significant interactions between main effects in ANOVAs of experiment 2 were examined using Tukey’s post hoc comparisons. In experiment 3, a 2 × 2 ANOVA analysis was used to analyze elevated plus maze behavior. Unpaired, two-tailed Student’s *t* tests were used to analyze the differences between saline-treated and heroin-treated animals within groups in the EPM as well as the stretch attend posture behaviors. In experiment 4, IL-1β, TNF-α, and GFAP immunoreactivity and cell counts were analyzed using an unpaired, two-tailed Student’s *t* test.

## Results

### Experiment 1: chronic escalating heroin administration produces withdrawal in females

The chronic escalating heroin administration (Fig. 1a) produced withdrawal behaviors 24 h after the last heroin dose in female rodents. χ^2^ analysis revealed no difference between the heroin withdrawal and saline controls for rearing behavior (*χ*^2^_(1,*n* = 23)_ = 0.144, *p* = 0.704). Importantly *χ*^2^ analysis revealed significant increases in withdrawal behaviors between the heroin withdrawal and saline controls for wet dog shakes (*χ*^2^_(1,*n* = 23)_ = 4.915, *p* = 0.027), teeth chattering (*χ*^2^_(1,*n* = 23)_ = 4.915, *p* = 0.027), diarrhea (χ^2^_(1,*n* = 23)_ = 6.244, *p* = 0.012), and weight loss (*χ*^2^_(1,*n* = 23)_ = 23, *p* < 0.001) (Fig. 1b). A 2 × 7 repeated measures ANOVA revealed a significant effect of day treatment on animal body weight (*F*_(6, 120)_ = 28.824, *p* < 0.001) (Fig. 1c). Student’s *t* test revealed no significant difference between groups on days 1–10 (*p* > 0.05), indicating no pre-existing weight differences. Additionally, both heroin and saline animals gained weight relatively similarly. Student’s *t* test revealed a significant difference between heroin and saline animals on day 11 (*t*_(20)_ = 2.618, *p* = 0.016), revealing heroin animals lost weight following the 24-h withdrawal in comparison to female animals. Student’s *t* test comparing weight change between day 10 and day 11 (first 24 h of withdrawal) revealed that heroin-treated animals lost significantly more weight than saline-treated animals (*t*_(21)_ = 7.212, *p* < 0.001) (Fig. 1d). A 2 × 2 ANOVA comparing sexes revealed a main effect of drug (*F*_(3,28)_ = 102.418, *p* < 0.001), but no main effect of sex (Fig. 1e), indicating that there were no sex differences in weight loss from heroin withdrawal.

**Fig. 2:**
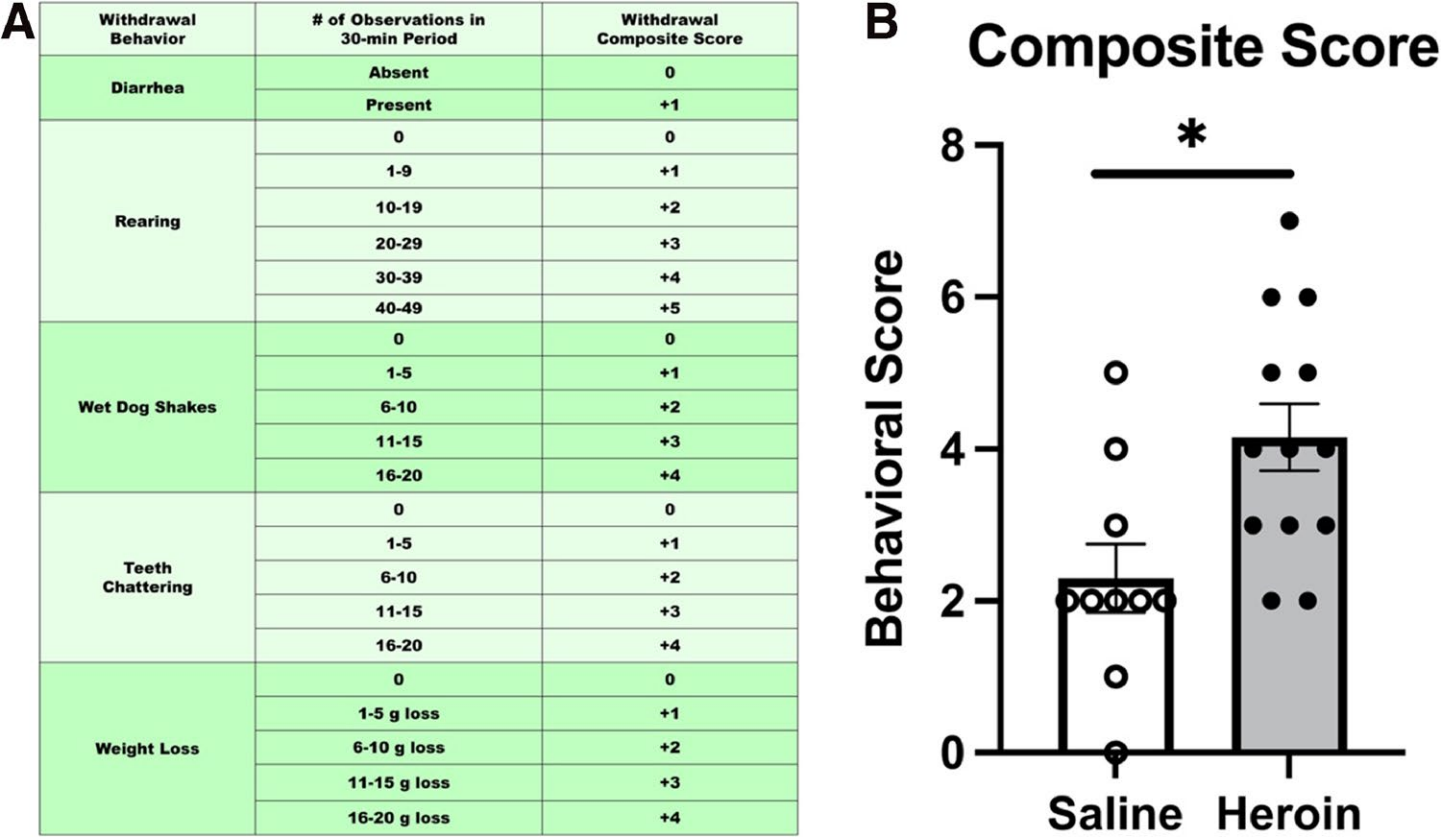
Chronic escalating heroin administration produced a higher composite withdrawal score in females. Withdrawal composite score explanation chart (**A**). Heroin-treated animals have a higher composite score (*N* = 23, *n* = 10–13) (**B**). *, statistically significant difference relative to respective control. Error bars indicate SEM

Student’s *t* test comparing composite withdrawal scores between heroin-treated animals and saline-treated animals revealed that heroin-treated animals had a significantly higher composite withdrawal score than saline-treated animals (*t*_(21)_ = − 2.922, *p* = 0.008) (Fig. 2b). These results indicate that chronic escalating heroin administration produces withdrawal in female animals that is comparable to males (see (Parekh et al. 2020) for male data).

### Experiment 2: heroin withdrawal does not produce enhanced fear learning in females

**Fig. 3:**
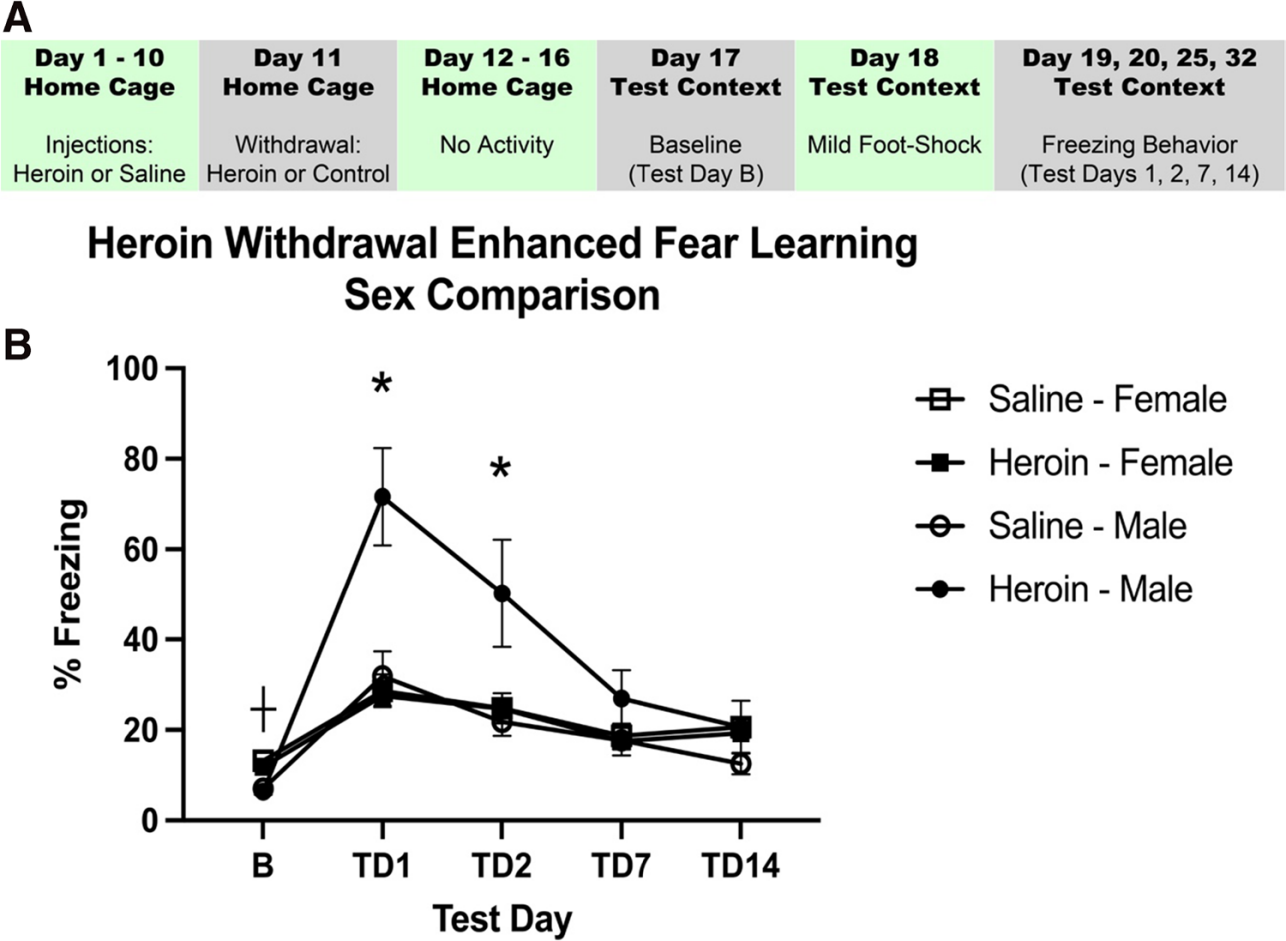
Heroin withdrawal does not cause future enhanced fear learning in females, but does in males. Experimental timeline (**A**). Heroin withdrawal does enhance contextual freezing behavior to a single mild foot shock in male animals but not in female animals (*N* = 36, *n* = 6–13) (**B**). *, statistically significant difference relative to respective control. + , statistically significant difference between sexes. Error bars indicate SEM

Heroin withdrawal produced subsequent enhanced fear learning in males and not in females. A two-way (sex, drug) ANOVA was used to analyze baseline freezing. There was no main effect of heroin treatment on baseline contextual freezing (*F*_(1,31)_ = 1.425, *p* = 0.242), indicating that there was no heroin-produced generalized fear to the novel context. There was a main effect of sex (*F*_(1,31)_ = 49.355, *p* < 0.001), indicating that male animals had a lower baseline freezing to the novel context than females. To avoid stress to female rodents induced by male pheromones, the sexes were run at different times, so slight differences in baseline freezing can be expected. However, all animals had below the 20% freezing threshold for baseline. A 2 (sex) × 2 (drug) × 4 (test day) repeated measures ANOVA was used to analyze test days and revealed a significant main effect of heroin treatment (*F*_(1,32)_ = 10.380, *p* = 0.003) and a significant main effect of sex (*F*_(1,32)_ = 7.932, *p* = 0.008). These main effects of heroin treatment and sex were on contextual freezing. There was also a significant effect of test day (*F*_(3,96)_ = 45.512, *p* < 0.001), indicating that conditioned freezing behavior diminished over time. Importantly, there was a significant heroin treatment by sex interaction (*F*_(1,32)_ = 12.333, *p* = 0.001). Tukey’s post hoc comparisons revealed heroin-treated males exhibited significantly higher freezing behaviors to saline-treated males and all female animals on test days 1 and 2 (*p* < 0.05) (Fig. 3b). These results replicate our previous findings in males and indicate sex differences between males and females in enhanced fear learning following heroin withdrawal.

**Fig. 4:**
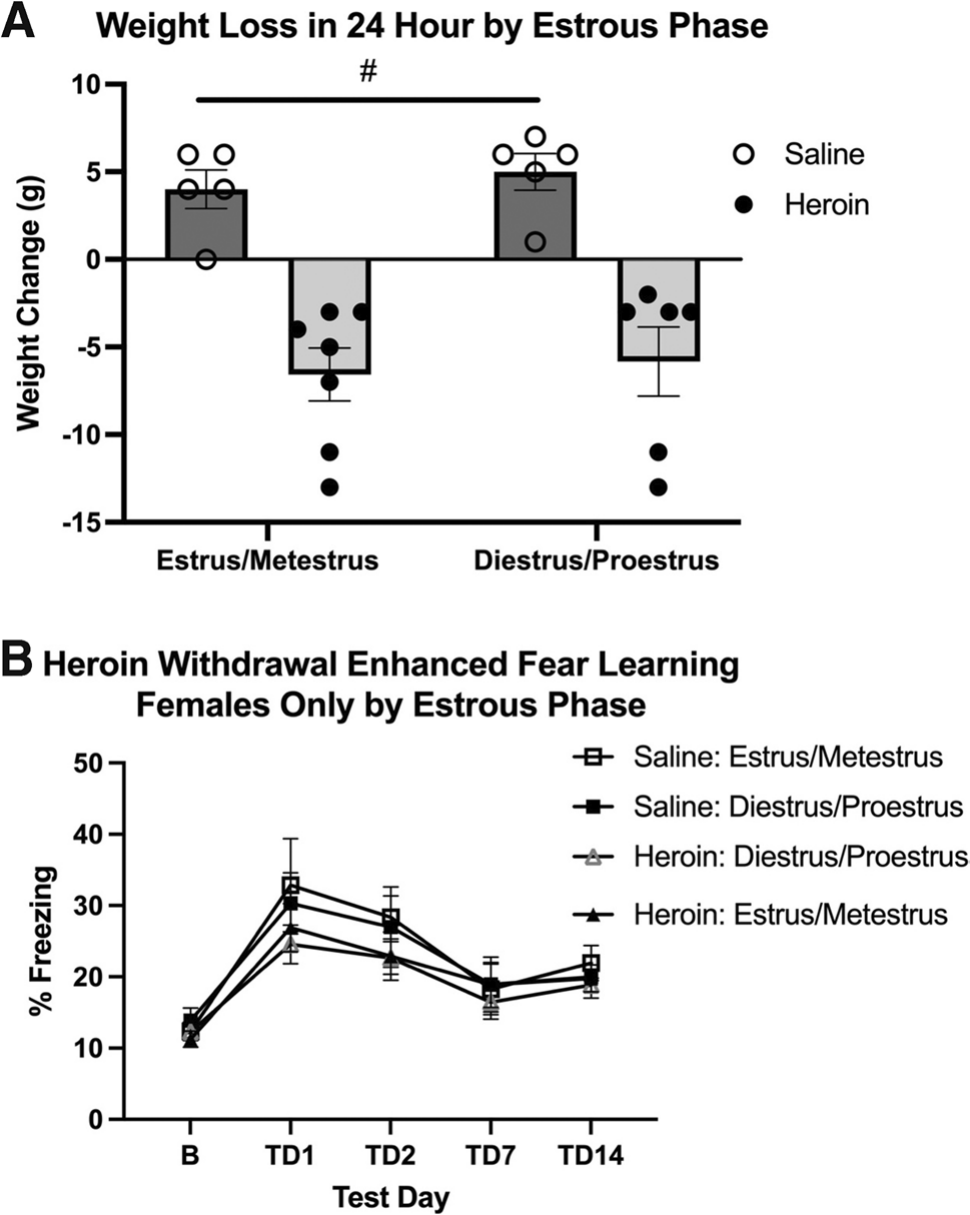
Estrous phase has no effect on weight loss in 24 h or future enhanced fear learning in females. Data from weight-loss females rats separated into low or increasing/high estradiol groups. Regardless of estrous distinction, heroin withdrawal significantly increased weight loss (*N* = 23, *n* = 5–7) (**A**). Data from enhanced fear learning rats separated into low or increasing/high estradiol groups. Regardless of estrous distinction, enhanced fear learning was not significantly different (*N* = 23, *n* = 5–7) (**B**). #, statistically significant main effect of heroin. Error bars indicate SEM

Because naturally cycling females demonstrated heroin withdrawal behaviors and did not demonstrate enhanced fear learning, estrous phase was determined for each sample and categorized into diestrus, proestrus, estrus, or metestrus (Fig. 4). The final analysis of withdrawal behaviors and enhanced fear learning (*n* = 8–12) when split across stage did not yield a large enough sample size to probe effects of estrous on heroin withdrawal behaviors and enhanced fear learning. Therefore, in experiments 1 and 2, samples in diestrus and proestrus were characterized as “increasing/high estradiol,” while those in estrus and metestrus were charactered as “low estradiol” (Santmyire et al. 2010; Miller and Takahashi 2013). In experiment 1, when weight loss was characterized by estrous phase, a 2 × 2 ANOVA revealed no significant effect of cycle (*p* > 0.05), indicating that estrous cycle played no role in the female heroin withdrawal behaviors. There was also a significant effect of drug (*F*_(3,19)_ = 16.047, *p* < 0.001), indicating that heroin-withdrawn animals still lost weight (Fig. 4a). In experiment 2, when freezing in enhanced fear learning was characterized by estrous phase, a two-way (drug, estrous cycle) ANOVA was used to analyze baseline freezing, and this revealed no main effect of cycle on baseline contextual freezing (*p* > 0.05), indicating that there is no effect of estrous cycle on baseline freezing. Additionally, 2 (drug) × 2 (estrous cycle) × 4 (test day) repeated measures ANOVA revealed no significant main effects of heroin treatment (*p* > 0.05) or estrous cycle (*p* > 0.05), indicating that there is no effect of estrous cycle on contextual freezing. There was a significant effect of test day (*F*_(3,13)_ = 5.624, *p* = 0.034), indicating that conditioned freezing behavior diminished over time (Fig. 4b). These results indicate that estrous cycle did not influence heroin withdrawal or future enhanced fear learning.

### Experiment 3: heroin withdrawal produces short-term anxiety in both males and females, but only produces long-term anxiety in males

**Fig. 5:**
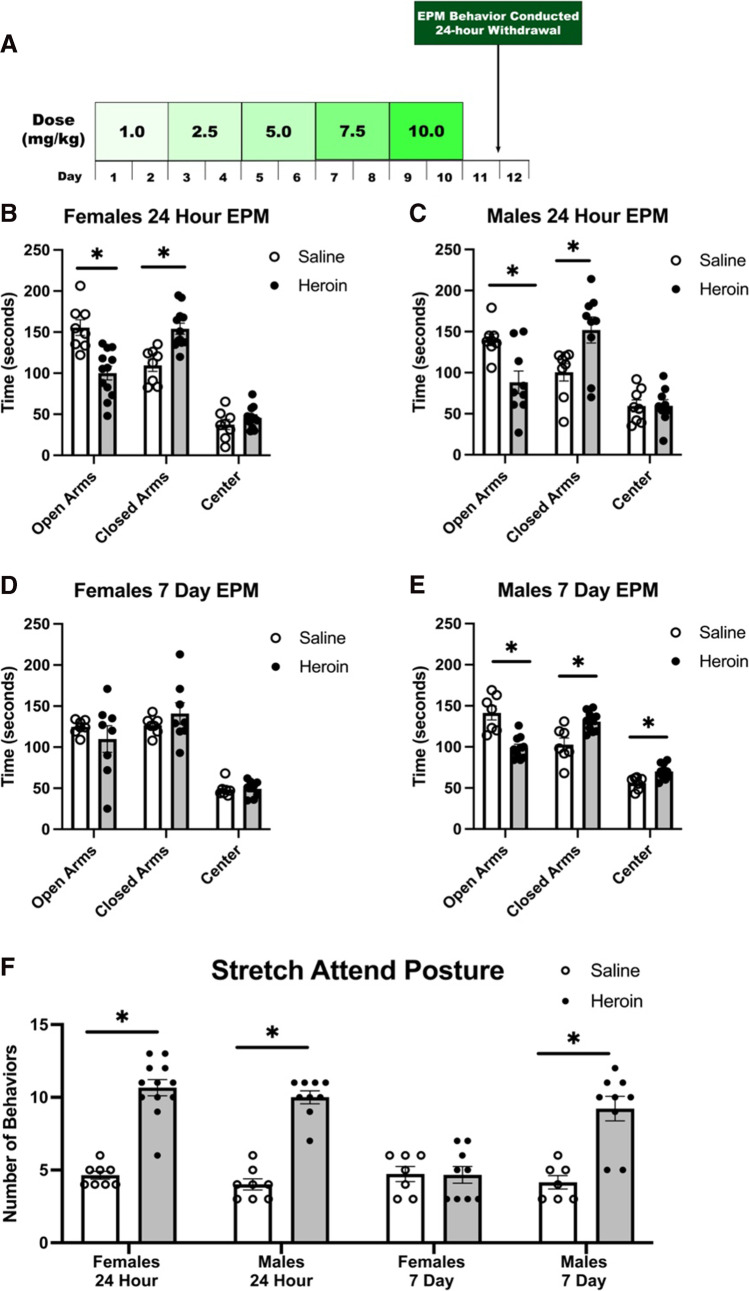
Heroin withdrawal produces short-term anxiety in both males and females but long-term anxiety in males, but not females. Experimental timeline (**A**). Twenty-four-hour female EPM (*N* = 20, *n* = 8–12) (**B**). Twenty-four-hour male EPM (*N* = 17, *n* = 8–9) (**C**). Seven-day female EPM (*N* = 15, *n* = 7–8) (**D**). 7-day male EPM (*N* = 17, *n* = 7–10) (**E**). Stretch attend posture behaviors across all times (*N* = 30, *n* = 7–8) (**F**). *, statistically significant difference relative to respective control. Error bars indicate SEM

Both male and females displayed elevated levels of short-term anxiety in the elevated plus maze 24-h following heroin withdrawal. A two-way ANOVA was performed to analyze the effect of drug and sex on time spent in the open arms, closed arms, and center. *t* tests were used to analyze the differences between saline-treated and heroin-treated animals within groups. Twenty-four hours following heroin administration, two-way ANOVA analysis revealed a significant main effect of drug in the open arms (*F*(1,31) = 28.775, *p* < 0.001) and closed arms (*F*(1,31) = 21.636, *p* < 0.001. There was no main effect of sex in the open arms or closed arms, but a significant main effect of sex in the center (*F*(1,31) = 6.370, *p* = 0.017). Post hoc tests revealed, 24 h following heroin administration, female heroin-treated animals spent less time in the open arms (*t*_(18)_ = 4.310, *p* < 0.001), more time in the closed arms (*t*_(18)_ = − 4.329, *p* < 0.001), and an equal time in the center (*t*_(18)_ = − 1.238, *p* = 0.232) compared to their saline-treated counterparts (Fig. 5b). Similarly, 24 h following heroin administration, male heroin-treated animals spent less time in the open arms (*t*_(15)_ = 3.213, *p* = 0.006), more time in the closed arms (*t*_(15)_ = − 2.631, *p* = 0.019), and an equal time in the center (*t*_(15)_ = − 0.027, *p* = 0.979) compared to their saline-treated counterparts (Fig. 5c).

Only males displayed elevated levels of long-term anxiety in the elevated plus maze 7-days following heroin withdrawal. A two-way ANOVA was performed to analyze the effect of drug and sex on time spent in the open arms, closed arms, and center. *t* tests were used as post-hoc tests to analyze the differences between saline-treated and heroin-treated animals within groups. Seven days following heroin administration, two-way ANOVA analysis revealed a significant main effect of drug in the open arms (*F*(1,28) = 9.061, *p* = 0.005), closed arms (*F*(1,28) = 6.841, *p* = 0.014), and center (*F*(1,28) = 5.035, *p* = 0.033). There was a main effect of sex in the closed arms (*F*(1,28) = 4.384, *p* = 0.045) and center (*F*(1,28) = 18.486, *p* < 0.001). Post hoc tests revealed 7 days following heroin administration, the time spent in open arms (*t*_(13)_ = 0.841, *p* = 0.416), closed arms (*t*_(13)_ = − 0.976, *p* = 0.347), and center (*t*_(13)_ = − 0.107, *p* = 0.917) was not significantly different in female heroin-treated animals and saline-treated animals (Fig. 5d). However, 7 days following heroin administration, male heroin-treated animals spent more time in the open arms (*t*_(15)_ = 4.880, *p* < 0.001), less time in the closed arms (*t*_(15)_ = − 3.504, *p* = 0.003), and more time in the center (*t*_(15)_ = − 3.309, *p* = 0.005) compared to their saline-treated counterparts (Fig. 5e). These results indicate that heroin withdrawal produces short-term anxiety in both males and females but only long-term anxiety in males.

Similarly, the stretch-attend posture behavior demonstrated this as well. *t* tests revealed 24 h following heroin administration, both heroin-treated females (*t*_(16)_ = − 12.059, *p* < 0.001) and males (*t*_(13)_ = − 12.953, *p* < 0.001) exhibited significantly higher behavior counts than their saline-treated counterparts. Seven days following heroin administration, heroin-treated females and saline-treated females had no significant difference in behaviors (*t*_(12)_ = 0.178, *p* = 0.862); however, heroin treated males exhibited significantly higher behavior counts than their saline-treated counterparts (*t*_(11)_ = − 9.797, *p* < 0.000) (Fig. 5f). This further indicates a sex difference in long-term anxiety following chronic escalating administration and withdrawal.

### Experiment 4: heroin withdrawal does not produce increased IL-1β, TNF-α, and GFAP immunoreactivity in females

**Fig. 6:**
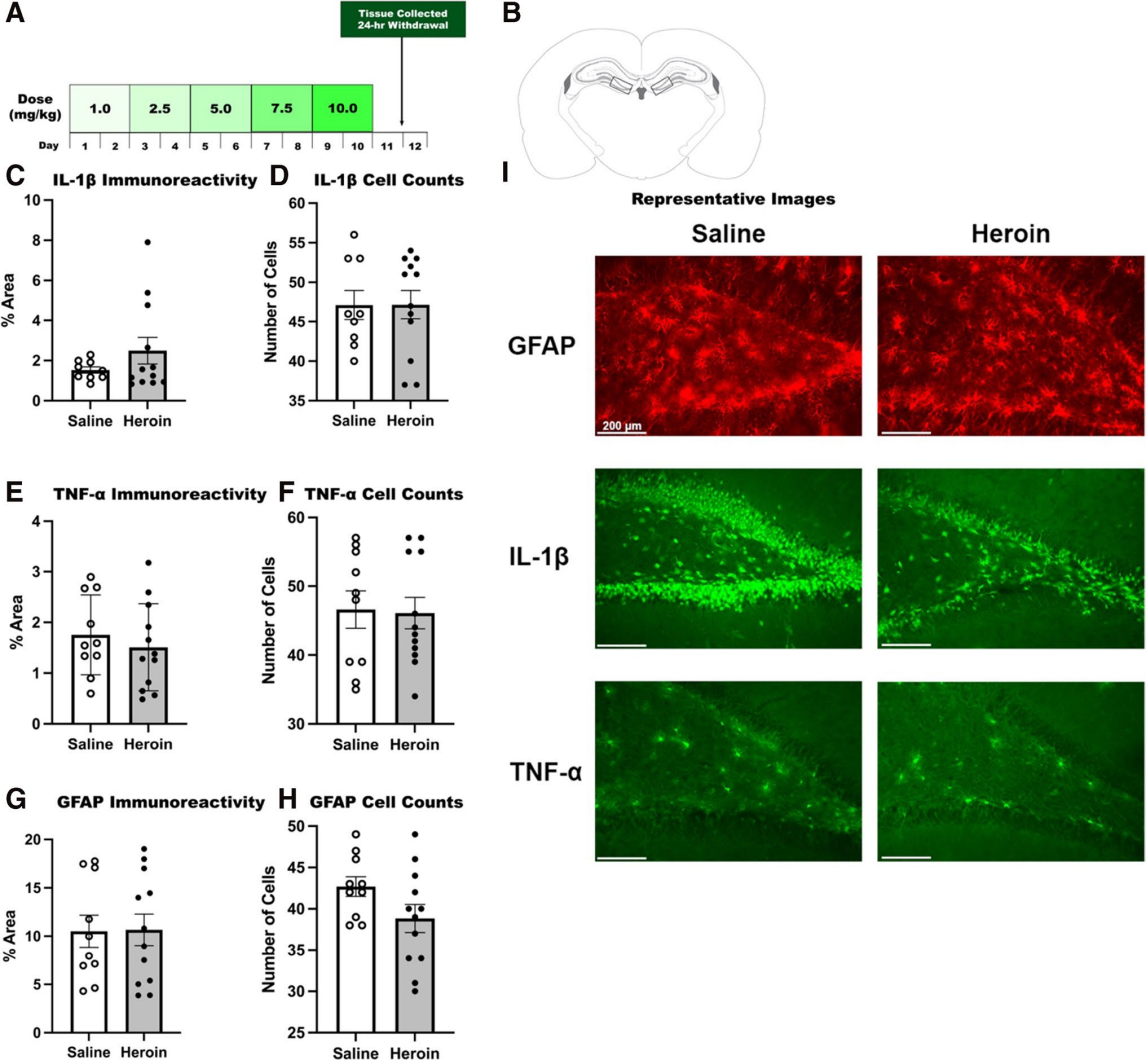
Twenty-four-hour heroin withdrawal does not increase IL-1β, TNF-α, and GFAP immunoreactivity in the dentate gyrus of the dorsal hippocampus in females. Experimental timeline (**A**). Paxinos and Watson (2007) schematic depicting bilateral image acquisition location. Each rectangular box represents the area in which images were taken within the DG of DH (**B**). Quantification of positive fluorescence stain (left) and cell counts (right) of IL-1β (*N* = 21, *n* = 9–12) (**C**, **D**), TNF-α (*N* = 22, *n* = 10–12) (**E**, **F**), and GFAP (*N* = 22, *n* = 10–12) (**G**, **H**). Representative images (× 20) for saline and heroin animals at the 24-h withdrawal timepoint (**I**). *, statistically significant difference relative to respective control. Error bars indicate SEM

IL-1β, TNF-α, and GFAP immunoreactivity was not increased following heroin withdrawal in females in the dentate gyrus of the dorsal hippocampus. Specifically, we focused on the DG subregion in the DH, as this is where we observed increased expression in males during heroin withdrawal (Parekh et al. 2020; Parekh et al. 2021). IL-1β immunoreactivity (*t*_(19)_ = − 1.232, *p* = 0.233) and cell counts (*t*_(19)_ = − 0.021, *p* = 0.983) were not significantly enhanced by heroin withdrawal in females (Fig. 6c, d). Additionally, TNF-α immunoreactivity (*t*_(20)_ = 0.692, *p* = 0.497) and cell counts (*t*_(20)_ = 0.147, *p* = 0.885) were not significantly enhanced by heroin withdrawal in females (Fig. 6e, f). Likewise, GFAP immunoreactivity (*t*_(20)_ = − 0.065, *p* = 0.949) and cell counts (*t*_(20)_ = 1.785, *p* = 0.089) were not significantly enhanced by heroin withdrawal in females (Fig. 6g, h). These results indicate sex differences between males and females in immunoreactivity and cell counts following heroin withdrawal (see (Parekh et al. 2020) for male data).

## Discussion

The current study demonstrates for the first time that chronic escalating heroin administration does not produce heroin withdrawal-induced EFL in female rats, as we have previously shown in male rats, but is capable of producing withdrawal behaviors in females that are similar to those of males. Additionally, heroin withdrawal produces short-term anxiety in both males and females, but only produces long-term anxiety in males. Following escalating heroin withdrawal, we did not observe increased immunoreactivity of IL-1β, TNF-α, or GFAP in the dentate gyrus of the dorsal hippocampus in female rats as previously observed in males. Additionally, estrous cycle stage did not have an effect on freezing behavior in females. These findings provide novel evidence that females may have differential fear-learning and anxiety responses to stress following chronic heroin administration and withdrawal.

Excitingly, chronic escalating heroin administration produced withdrawal behaviors in female rodents 24 h following the last heroin injection. At this withdrawal time point, heroin-treated animals showed significant increases in withdrawal behaviors as compared to the saline-treated animals and lost significantly more weight than saline-treated females. Importantly, these female withdrawal behaviors are comparable to the male animals. This is consistent with the literature, as most signs of spontaneous opioid withdrawal in male and females are symptomatically similar in preclinical (Gipson et al. 2021) and clinical (Back et al. 2011) populations. Since symptom severity is similar across male and female rats, there were no sex differences in withdrawal severity. In turn, we would predict that female rats would also demonstrate enhanced fear learning and similar neuroinflammatory markers following heroin withdrawal, as previously demonstrated in male rats (Parekh et al. 2020; Parekh et al. 2021). However, female rats exposed to heroin withdrawal did not show enhanced fear learning, or increased immunoreactivity of IL-1β, TNF-α, or GFAP.

Overall, it may seem that female’s lack of freezing behavior and pro-inflammatory cytokine immunoreactivity suggests resilience to the severe effects of heroin withdrawal. However, other measures may indicate that females are not resilient, but simply respond differently to trauma. For example, a differential trauma response in females rather than resilience to trauma is supported by pre-clinical findings demonstrating that trauma-exposed male rats display more physiological sensitivity, such as an enhanced acoustic startle response and exaggerated negative feedback control of the hypothalamic–pituitary–adrenal axis (Albrechet-Souza et al. 2020; Knox et al. 2021). This is consistent with the enhanced freezing demonstrated by the male rats in our study. The clinical literature also depicts a similar incongruence in symptomatic presentation between males and females, with women showing higher anxiety levels in cognitive and affective dimensions compared to men, but not in physiological and autonomic dimensions (Tolin and Foa 2006). This amplified male physiological sensitivity would explain the elevated freezing we observed in males but not females, as hyperarousal is well-captured by the EFL paradigm.

Based on these findings, the EFL model used in these experiments to examine the animals’ trauma response captures clinical symptoms of hyperarousal, as animals show sensitized reactions to stimuli that are perceived as threatening. It is possible that the EFL model may better capture physiological symptoms of PTSD rather than affective symptoms, and the females’ lack of response in the EFL paradigm may be an indication that this model does not effectively reproduce female symptomatic presentation of PTSD. It is not known whether the presentation of a trauma response is different between males and females or if females have differing sensitivities to various types of stressors. The foot shock stressor in the EFL paradigm has a strong physiological component. Other models using a strong psychological stressor, such as the predator odor avoidance model or chronic variable stress model, may create a stronger effect in females rather than males. Further studies encompassing different animal models are needed to tease out the symptomatic sex differences in trauma response.

Given the sex differences in neuroinflammatory markers we observed, it is possible that immunoreactivity of IL-1 and TNF-α in the dorsal hippocampus may only underlie the male fear response, so a lack of neuroinflammation in females does not necessarily indicate resilience to heroin withdrawal. Neuroanatomical and morphological differences between males and females may also explain the differences in immunoreactivity we observed. For example, sex-differences in astrocyte functioning could also account for these immunological differences (Cui et al. 2014). Astrocytes play a role in innate immune responses by producing cytokines and chemokines such as IL-1β and TNF-α (Zhang et al. 2018). In the dorsal hippocampus, male rats have more GFAP immunoreactive astrocytes (Conejo et al. 2003). Because GFAP expression is a marker for astrocyte reactivity, increased astrocyte reactivity in males could explain enhanced immunoreactivity of IL-1β and TNF-α following heroin withdrawal in males. Additionally, males have also been shown to have a larger dentate gyrus and greater synaptic connectivity than females (Tabibnia et al. 1999). Due to this increased size and connectivity, it is possible that the dentate gyrus is a larger mediator of maladaptive fear responses in males, whereas other regions and pathways may play a greater role in the female fear-learning response. For example, although we did not observe an inflammatory response in the dentate gyrus in females, male rats have been shown to have higher basal levels of inflammatory mediators in the hippocampus and cortex, whereas females have higher inflammatory mediators in the amygdala (Nelson and Lenz 2017). In turn, it is possible that the amygdala may play a larger role in the female response to heroin withdrawal, providing an additional area of examination for further studies of PTSD/OUD comorbidity in females.

Sex-mediated differences in IL-1β regulation could also account for the differences in IL-1β immunoreactivity we observed following heroin withdrawal. For example, stress exposure affects the regulation of brain IL-1β in males (Laryea et al. 2013), but not females, and adolescent stress increased hippocampal inflammatory responses to LPS in males only (Lin et al. 2009). Additionally, sex-differences in gene expression could also explain immunoreactivity differences. Following trauma, there are sex-differences in cFos activation and glucocorticoid receptor expression in the brain, which may underlie sex-differences in trauma response behaviors. IL-1β and TNF-α both induce cFos activation (Hermann et al. 2001). The elevated inflammatory cytokines we observed following heroin withdrawal in male rats, but not female rats, may be a possible mechanism for this divergent gene expression, and subsequent symptomatic divergence in freezing behavior. Further, inflammation can also mediate glucocorticoid receptor expression, which regulates HPA axis activity during the stress response. Interestingly, a loss of glucocorticoid receptors results in severe HPA axis hyperactivity (Laryea et al. 2013), and both IL-1β and TNF-α exposure has been shown to reduce glucocorticoid receptor gene levels and cytoplasmic levels (Escoll et al. 2015; Dendoncker et al. 2019). These findings suggest that a stress-induced, neuroinflammatory response may also underlie male’s glucocorticoid receptor expression changes and associated HPA axis dysfunction. Overall, the literature suggests that neuroinflammatory responses may be related to male-specific trauma responses, whereas other inflammatory mediators and brain regions may mediate the female trauma response.

The current experiments utilized naturally cycling female rats and examined estrous stage at major time points such as 24-h withdrawal, baseline, mild shock, and all test days in the enhanced fear learning paradigm. There appeared to be no effect of estrous cycle on either heroin withdrawal or enhanced fear learning as all heroin-withdrawn females demonstrated withdrawal behaviors and similar freezing levels. When animals were separated across estrous stage, there was insufficient sample size to adequately assess if withdrawal behaviors and freezing levels varied across the four stages of estrous cycle. Therefore, two stages were combined, and animals were characterized as either increasing/higher estradiol (diestrus and proestrus) or low estradiol (estrus and metestrus) stages. We demonstrate in the current studies that females in both the high and low estradiol phases exhibit heroin withdrawal behaviors as well as freezing.

Although we did not see any evidence of enhanced cytokine production in females following heroin withdrawal, or an effect of estrous cycle on heroin withdrawal behaviors or enhanced fear learning, other immune measures may be modified by female sex hormones. The current study was optimized to maximally detect changes in heroin withdrawal-induced cytokines especially in the dorsal hippocampus, as this is a direct comparison to our previous work in males (Parekh et al. 2020; Parekh et al. 2021). Collectively, our results are the first to demonstrate sex differences in cytokine production following heroin withdrawal in female rats. To examine whether heroin withdrawal effects extend to other pro-inflammatory agents or differ based on heroin doses and/or frequency, future experiments can alter the heroin paradigm to explore the interaction of heroin administration, heroin withdrawal, sex hormones, and cytokine production in female rats. Additionally, in these experiments estrous stages were grouped based on characteristic levels of estradiol across cycle. This did not account for the effect of other female sex hormones on withdrawal behaviors or enhanced fear learning, such as progesterone. Future experiments can examine whether female sex hormones, including both estradiol and progesterone, directly modulate the mechanisms regulating heroin withdrawal and future enhanced fear learning.

While enhanced fear learning is a subsequent behavior following heroin withdrawal, generalized anxiety is also a common symptom of heroin withdrawal. Since we observed sex-differences in stress enhanced fear learning, we next assessed generalized anxiety sex differences following heroin withdrawal. We measured the anxiety response using the elevated plus maze assay (EPM) and found that heroin withdrawal produces short-term anxiety in both males and females (observed at the 24-h time point), but surprisingly, only produces long-term anxiety in males (assessed at the 7-day time point). We predict that the two anxiety behaviors we observe following withdrawal (enhanced fear learning and generalized anxiety) are mediated by separate, distinct biological mechanisms and neural circuitry than fear learning behaviors. In turn, alterations in hippocampal cytokine levels may not be related to the enhanced generalized anxiety response, and may instead be mediated by other mechanisms, such as the reward system. For example, the central nucleus of the amygdala is well-established in increasing reward thresholds, anxiety-like responses, and extracellular levels of corticotropin-releasing factor during opioid withdrawal (Back et al. 2011; Logrip et al. 2011; Koob and Volkow 2016). Sex differences in brain-derived neurotrophic factor (BDNF) and cyclic adenosine monophosphate response element–binding protein (CREB) in the amygdala may also underlie the male long-term anxiety response. In the amygdala, males are shown to have significant decreases in BDNF and CREB following stress, which are implicated in the anxiety response and could contribute to chronic anxiety following the stress of opioid withdrawal (Lin et al. 2009). To examine whether the reward-system may account for differences in the anxiety behaviors we observed, future experiments can examine sex differences in other brain regions such as the amygdala. Additionally, trauma-exposed male rats are shown to display more physiological sensitivity which may explain their enhanced anxiety response (Albrechet-Souza et al. 2020; Knox et al. 2021).

In summary, our exciting findings demonstrate that heroin administration and withdrawal produce withdrawal behaviors in females that are similar to those of males. However, chronic escalating heroin administration does not produce enhanced fear learning in female rats or increased immunoreactivity of IL-1β, TNF-α, and GFAP in the dentate gyrus of the dorsal hippocampus in female rats as previously seen in males. Additionally, heroin withdrawal produces short-term anxiety in both males and females, but only produces long-term anxiety in males. Collectively, these data provide important new evidence of sex differences following heroin withdrawal and evidence that females may have a differential fear-learning and anxiety response to stress. The current study demonstrates the need to further study sex differences in both drug and trauma responses to uncover the neurobiological mechanisms underlying sex differences in symptoms.

## Data Availability

Raw data from published studies is available to investigators upon request.

## Declarations

### Welfare of animals statement

The studies conducted in this manuscript adhere to legal and institutional animal welfare guidelines. All procedures were conducted with approval from the University of North Carolina at Chapel Hill Institutional Animal Care and Use Committee.
